# Dynamic Effects Arise Due to Consumers’ Preferences Depending on Past Choices

**DOI:** 10.3390/e22020173

**Published:** 2020-02-03

**Authors:** Sameh S. Askar, A. Al-khedhairi

**Affiliations:** 1 Department of Statistics and Operations Research, College of Science, King Saud University, P.O. Box 2455, Riyadh 11451, Saudi Arabia; akhediri@ksu.edu.sa; 2 Department of Mathematics, Faculty of Science, Mansoura University, Mansoura 35516, Egypt

**Keywords:** logistic map, Cobb–Douglas, duopoly, stability, chaotic attractor, bifurcation, chaos, entropy

## Abstract

We analyzed a dynamic duopoly game where players adopt specific preferences. These preferences are derived from Cobb–Douglas utility function with the assumption that they depend on past choices. For this paper, we investigated two possible cases for the suggested game. The first case considers only focusing on the action done by one player. This action reduces the game’s map to a one-dimensional map, which is the logistic map. Using analytical and numerical simulation, the stability of fixed points of this map is studied. In the second case, we focus on the actions applied by both players. The fixed points, in this case, are calculated, and their stability is discussed. The conditions of stability are provided in terms of the game’s parameters. Numerical simulation is carried out to give local and global investigations of the chaotic behavior of the game’s map. In addition, we use a statistical measure, such as entropy, to get more evidences on the regularity and predictability of time series associated with this case.

## 1. Introduction

As it is known in economy that the Cobb–Douglas utility function is emanated from empirical production studies. It was commonly used in different areas of macroeconomics and forecast production to organize production data. It is not an assumed function, but it is derived from demand theory as cited in [1]. It is a nonlinear function of demand, and it is more suitable in studying dynamic economic games based on it than those functions which are linear and exponential. It was the core of several studies of dynamic economy, such as oligopoly games. Here, we give some cited dynamic studies that were constructed based on Cobb–Douglas utility function. For instance, this utility function was adopted in Reference [2] for studying the dynamic game between four competed firms. In Reference [3], the authors studied a specific game where a private good is formed for identical competed firms under some technologies represented by the production function of Cobb–Douglas. In Reference [4], a heterogeneous duopoly game constructed based on an isoelastic demand function derived from Cobb–Douglas utility function was analyzed. It was also used in studying the dynamic characteristics that arise in a fish stock harvested game consisting of two competitors with cost functions derived from it in Reference [5]. A generalized form of this utility function was adopted in Reference [6] in order to study an oligopoly game where players require renewal of their capital equipment. In [7], a duopoly competed game with capacity constraints was investigated using a production function stipulated from Cobb–Douglas. With Cobb–Douglas preferences, a Cournot duopoly game was introduced and studied in Reference [8]. Another Cournot duopoly game with competed players having capacity constraints was considered based on utility function of Cobb–Douglas in Reference [9]. Other studies for Cobb–Douglas and the dynamic analysis for games based on it can be found elsewhere [10,11,12,13,14,15,16]. In Reference [17], the complex dynamic characteristics of a novel nonlinear finance system was investigated. A fractional finance system in which parameters are negative values was introduced and discussed in Reference [18]. In Reference [19], a proposed approach was introduced to detect computer simulation that is unreliable.

In the current manuscript, we recall the form of Cobb–Douglas utility function in order to build our model. We consider that the competitors only concern preferences that depend on past choices. This means that the output elasticity of the Cobb–Douglas utility function is not constant and depends on past choices of demand. So, our contribution in this paper is to investigate the influences of such non-constant preferences on the economic model constructed based on that. Moreover, the main novelty of our discussed models with respect to other models that exist in literature includes multi-stability of different types of periodic cycles in the numerical simulation. This assumption yields a nonlinear, two-dimensional discrete dynamic map describing our models in this manuscript. However, this map is simple, but its dynamic characteristics show two types of bifurcation which destabilize its fixed points. The main results of the proposed work concern the local stabilization of the fixed points; the investigation of the stability conditions which identify the routes where the fixed points lose their stabilities; the study of global characteristics of the game’s map; and discussion of the basin of attraction of high periodic cycles obtained due to those bifurcations. All the results in the numerical simulation sections are obtained using E and F chaos free software. More details about this software can be found in Reference [20].

Now, the current paper can be summarized as follows. In Section 2, we build our model by recalling the Cobb–Douglas utility function. In Section 3, we consider a case where the consumer focuses only on one good and ignores the other. This reduces the map to a one-dimensional map. It is a general type of the logistic map. Some numerical simulations are performed in this section to get more insights about the stability of fixed points of this logistic map. In addition, we investigate the stability of two-dimensional map in detail. This includes local and global analysis of the fixed points. Furthermore, we measure the entropy of time series of the map under certain parameter values. Finally, the conclusion is presented in Section 4.

## 2. The Model

The model begins by recalling the typical Cobb–Douglas utility function that is maximized subject to a budget constraint. Let *x* and *y* denote the consumption of two goods, *p* and *q* are their prices, respectively, and *m* is the individual’s level of income. Then, we have the following optimization problem,

(1) $$\begin{array}{c} Max\;U=x^{a}y^{1-a}\\ s.t.\;\;\;px+qy=m. \end{array}$$

Set up the Lagrange function as follow:

(2) $$L\left(x,y\right)=x^{a}y^{1-a}-\lambda \left(\;px+qy-m\right).$$

The first-order conditions lead to

(3) $$\begin{array}{c} \frac{\partial L}{\partial x}=\frac{aU}{x}-\lambda p,\\ \frac{\partial L}{\partial y}=\frac{(1-a)U}{y}-\lambda q,\\ \frac{\partial L}{\partial \lambda }=m-px-qy. \end{array}$$

These conditions give establishing the following demand curves,

(4) $$\begin{array}{c} x=\frac{m}{p}a,\\ y=\frac{m}{q}\left(1-a\right). \end{array}$$

The parameter *a* in the utility function represents a property of preferences. We assume here that this parameter depends endogenously on past choices. More specifically, we assume it takes the form, $a_{t+1}=bx_{t}y_{t}$. The parameter *b* denotes an experience-dependent parameter. Any increase in the parameter *b* will give an increase in the parameter *a* in the next period; hence, more preferences swing in favor of the good *x*. Using this assumption, one can get the following two dimensional map:

(5) $$\begin{array}{c} x_{t+1}=\frac{m}{p}bx_{t}y_{t},\\ y_{t+1}=\frac{m}{q}\left(1-bx_{t}y_{t}\right). \end{array}$$

## 3. Analysis of Map 5

### 3.1. Analysis of 1D-Map

First, we start our analysis by assuming that the consumer focuses only on the good denoted by $x_{t+1}$. Therefore, from the budget constraint, one can get the following one-dimensional map:

(6) $$x_{t+1}=\frac{mb}{pq}x_{t}\left(m-px_{t}\right),$$

which gives the logistic map. This map admits two fixed points, $\bar{x}=0,\bar{x}=\frac{bm^{2}-pq}{bmp}$. Since the consumption of the quantity *x* must be positive, we should restrict it by $bm^{2}>pq$. Here, we focus on the second fixed point because it gives a maximum consumption of the good *x*, as given in the following discussion.

**Proposition** **1.** *The maximum consumption of the good x should be restricted by the relation $pq<bm^{2}<4qp^{2}.$*

**Proof.**  Let $f\left(x\right)=\frac{mb}{pq}x\left(m-px\right)$; then, $f^{{}^{\prime }}\left(x\right)=0$ gives a maximum value $x=\frac{m}{2p}$, and the maximum consumption becomes $f\left(\frac{m}{2p}\right)=\frac{bm^{3}}{4qp^{2}}$. Then, the maximum consumption cannot exceed the total income *m*. So, we get $\frac{bm^{2}}{4qp^{2}}<1$. This gives the constraint $pq<bm^{2}<4qp^{2}$. □

**Proposition** **2.** *The solution $\bar{x}=\frac{bm^{2}-pq}{bmp}$ is asymptotically stable if $bm^{2}<3pq$.*

**Proof.**  It is stable if ${\left|\frac{df(x)}{dx}\right|}_{x=\bar{x}}<1$; then, this completes the proof. □

The stability of the point $\bar{x}=0$ is the same as in proposition 2.

#### Numerical Simulation (1D-Map)

**Figure 1:**
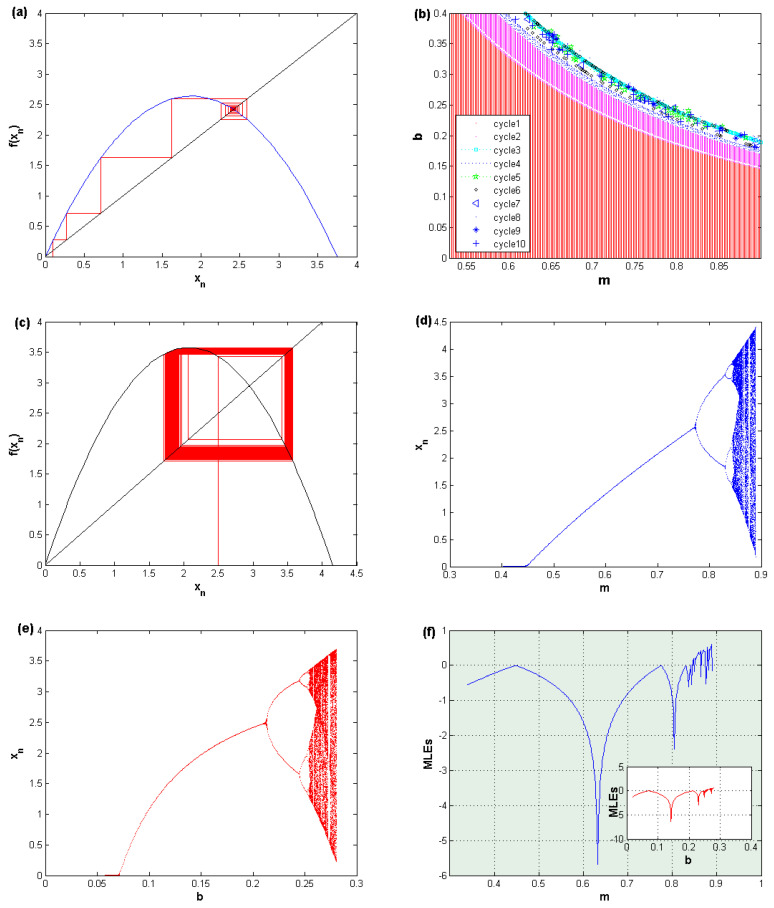
(**a**) The cobweb diagram for the stable fixed point; (**b**) 2D-Bifurcation diagram for the parameters *m* and *b*. (**c**) The cobweb diagram for the unstable fixed point. (**d**) Bifurcation diagram when varying the parameter *m*. (**e**) Bifurcation diagram when varying the parameter *b*. (**f**) Maximum Lyapunov exponents of *m* and *b*.

The above discussion shows that there are only two parameters which have an impact on the stability of the nonzero fixed point. These parameters are *m* and *b*. Assuming the following parameter values, m=0.75,b=0.2,p=0.2, and q=0.2, Figure 1a shows the cobweb diagram of the stable fixed point $\bar{x}=2.4167$. The figure shows that the iterations are converging well to the stable fixed point. After that, this fixed point loses its stability due to the construction of period doubling bifurcation. This period doubling bifurcation is formed due to the consequence of different types of periods. Figure 1b presents the relation between the two parameters *m* and *b*. It shows the regions where different period cycles can appear at the value parameters p=0.2, and q=0.2. At the value parameters m=0.83,b=0.2,p=0.2, and q=0.2 the stable fixed point becomes unstable. In Figure 1c, the cobweb map that provides the instability of the fixed point is given. As said previously, both the parameters *m* and *b* have an influence on the behavior of the map (6). Thus, Figure 1d shows that the fixed point of that map loses its stability due to the construction of period cycles, and then flip bifurcation arises. The same observation is given in Figure 1e, where the flip bifurcation is formed due to varying in the parameter *b*. Confirmation on the chaotic behavior of that map is given in Figure 1f. It presents the maximum Lyapunov exponent against the parameters *m* and *b*. Eventually, the fixed point of map (6) starts asymptotically stable and then becomes unstable due to an increase in both parameters *m* and *b*.

### 3.2. Analysis of the 2D-Map

Now, we study the map (5) on which the consumer focuses on both quantities. Analyzing this map requires first calculating its positive fixed points.

**Proposition** **3.** *The map (5) has two fixed points $e_{1}=\left(0,\frac{m}{q}\right)$ and $e_{2}=\left(\frac{bm^{2}-pq}{bmp},\frac{p}{bm}\right)$*

**Proposition** **4.** *The fixed point $e_{1}$ is locally stable if $\frac{m^{2}b}{pq}<1.$*

**Proof.**  The Jacobian matrix at this point takes the form, $J=\left(\begin{array}{cc} \frac{m^{2}b}{pq} & 0\\ -\frac{m^{2}b}{q^{2}} & 0 \end{array}\right)$, and then the characteristic equation yields the eigenvalues $\lambda_{1}=0,\lambda_{2}=\frac{m^{2}b}{pq}$. So, the point is locally stable if $\left|\lambda_{2}\right|<1$. □

**Proposition** **5.** *The fixed point $e_{2}$ is locally asymptotically stable if $bm^{2}<3pq$.*

**Proof.**  The Jacobian matrix at this point takes the form, $J=\left(\begin{array}{cc} 1 & \frac{bm^{2}-pq}{p^{2}}\\ -\frac{b}{q} & -\frac{bm^{2}-pq}{pq} \end{array}\right)$, and then the characteristic equation yields the eigenvalues $\lambda_{1}=0,\lambda_{2}=-\frac{m^{2}b-2pq}{pq}$. So, the point is locally asymptotically stable if $\left|\lambda_{2}\right|<1$. □

**Proposition** **6.** *The system (5) at the fixed point $e_{2}$ loses its stability due to two types of bifurcations: (i) Fold bifurcation if $bm^{2}=pq$ and (ii) Flip bifurcation if $bm^{2}=3pq$.*

**Proof.**  It is easy to get Jury conditions from the above Jacobian at $e_{2}$ as follows:

$$\begin{array}{c} 1+T\left(J\right)+D\left(J\right)=2-\frac{bm^{2}-pq}{pq},\\ 1-T\left(J\right)+D\left(J\right)=\frac{bm^{2}-pq}{pq},\\ 1-D(J)=1 \end{array}$$

where $T\left(J\right)=1-\frac{bm^{2}-pq}{pq}$ and D(J)=0 refer to the trace and determinant of the above Jacobian, respectively. If $bm^{2}=pq$, then both the first and the third conditions are positive, while the second condition equals zero. Therefore, one of the eigenvalue will equal 1, and then we get a fold bifurcation. On the other hand, if $bm^{2}=3pq$ this means that both the second and the third conditions will be positive, while the first condition equals zero, and then one of the eigenvalue will equal −1; hence, the stability no longer exists due to the appearance of flip bifurcation. □

#### 3.2.1. Numerical Simulation (2D-map)

**Figure 2:**
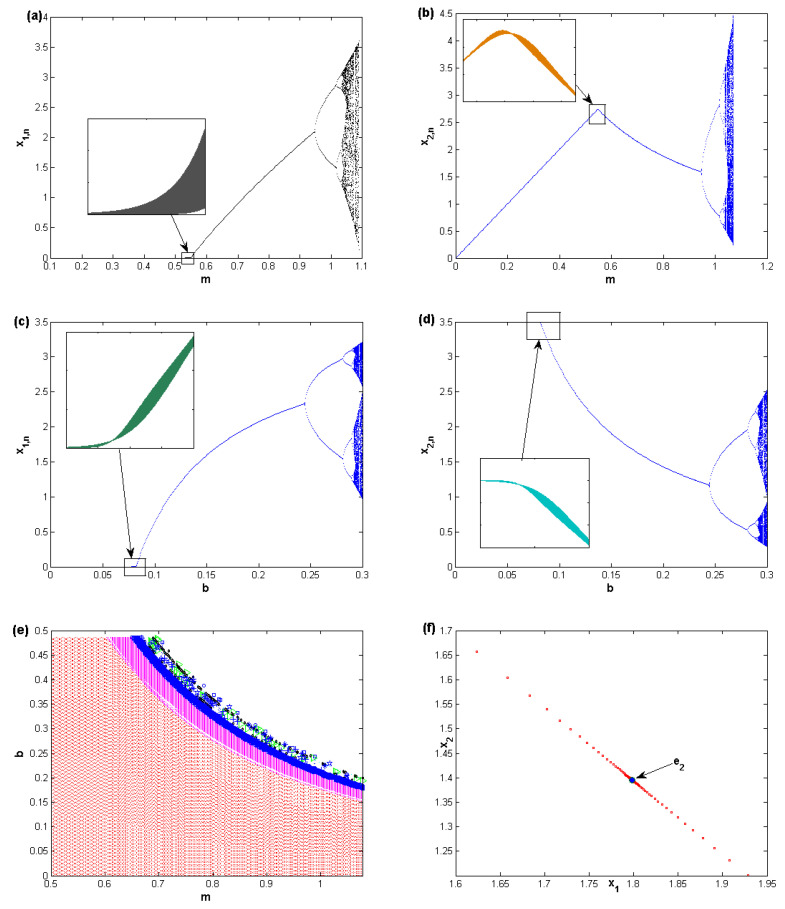
(**a**) Bifurcation diagram for $x_{1}$ when varying *m*. (**b**) Bifurcation diagram for $x_{2}$ when varying *m*. (**c**) Bifurcation diagram for $x_{1}$ when varying *b*. (**d**) Bifurcation diagram for $x_{2}$ when varying *b*. (**e**) 2D-Bifurcation diagram in the (m,b)-plane for the map (5). (**f**) The phase portrait of the stable fixed point $e_{2}$.

This section gives some insights about the results obtained in the previous section. Here, we use numerical simulation by assuming specific values by which we try to identify the region of the stability and the chaotic behavior of map (5). Assuming the parameter values, b=0.2,p=0.3, and q=0.2, with the initials $x_{1,0}=0.11$ and $x_{2,0}=0.12$, Figure 2 shows the bifurcation diagrams of the variables $x_{1}$ and $x_{2}$ when varying the parameter *m*. In Figure 2a,b, one can see that the map (5) loses its stability due to fold bifurcation first, followed by flip bifurcation. The other parameter, *b*, also has a chaotic influence on the map. Figure 2c,d show the bifurcation diagrams due to any increase in the parameter *b*. It also shows two types of bifurcations by which the map loses its stability. Those bifurcations are constructed at the same parameter values, b=0.2,p=0.3, and q=0.2, with the initials $x_{1,0}=0.11$ and $x_{2,0}=0.12$. Therefore, it is given in Figure 2e as a 2D-bifurcation diagram in the (m,b)-plane to show high periodic cycles that make the map enter the chaotic region. The red color in Figure 2e refers to the region where the fixed point is locally stable. The other colors refer to different types of periodic cycles. This urges us to investigate the behavior of this map more, as follows. Assuming the following parameter values, m=0.8186,b=0.2626, and p=0.3,q=0.2, gives a stable fixed point in the phase plane. It is clear in Figure 2f that the only stable point is the fixed point $e_{2}$. The figure gives the phase portrait of this stable fixed point. At those parameters, it is easy to see that 1+T(J)+D(J)=1.8260 and 1−T(J)+D(J)=1.933, which are both positive, and then the fixed point $e_{2}$ is stable. This local analysis may not provide us with some predictions on the future evolution of the complex characteristics of map (5). This is because of the simplicity of the local analysis and, in addition, because any local investigations regarding the local stability of the fixed point $e_{2}$ focus only on a small neighborhood around this point. Another reason is about the initial values of the decision variables on which they are not combined with those small neighborhoods in such investigations. This requires us to do global analysis on the behavior of the map (5) in order to get more confirmation on the long-run behavior of this map.

**Figure 3:**
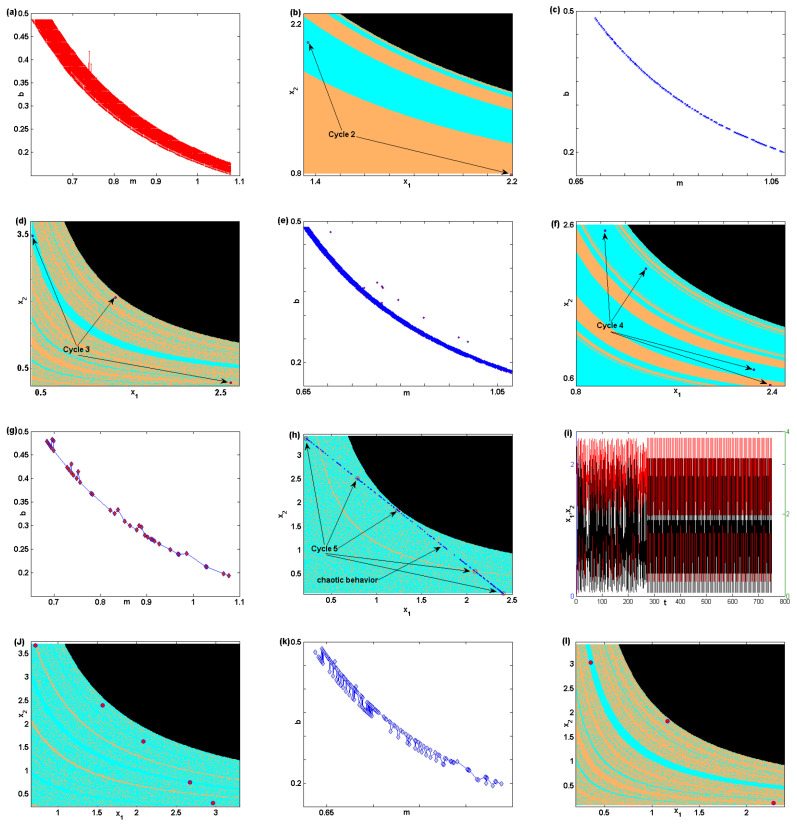
(**a**) The region of period 2-cycles. (**b**) The basin of attraction of period 2-cycle. (**c**) The region of period 3-cycles. (**d**) The basin of attraction of period 3-cycle. (**e**) The region of period 4-cycles. (**f**) The basin of attraction of period 4-cycle. (**g**) The region of period 5-cycles. (**h**) The basin of attraction of period 5-cycle. (**i**) Time series for the map’s variables at m=0.7372 and b=0.4312. (**j**) The basin of attraction of period 5-cycle. (**k**) The region of period 6-cycles. (**l**) The basin of attraction of period 3-cycle.

For more global investigations, we highlight more in the 2D bifurcation diagram in Figure 2e. This diagram can be divided into some sections where cycles with different periods are obtained. Starting with cycles of period 2, we plot Figure 3a where any point in this plot gives period 2-cycles. For instance, we assume m=0.8162 and b=0.2907, with the other parameter values fixed, and we get a stable cycle of period 2. It is given in Figure 3b with its basin of attraction, and the black color refers to divergent points. For the reader’s information, any suggested point in the region defined in Figure 3a will yield only a stable period of cycle 2. Figure 3c shows a region in the 2D bifurcation diagram on where cycles of period 3 exist. Numerical simulation shows that any point in this region will give only stable cycles of period 3. Making only an increase in the parameter *b* to the value 0.3458 and keeping the other parameters fixed, including *m*, we get a stable cycle of period 3. It is depicted in Figure 3d with its basin. Assuming b=0.3155, with the other parameter values fixed, the region where cycles with period 4 exist is given in Figure 3e. This region in the 2D bifurcation diagram includes only values of *m* and *b* where we can only get this type of periodic cycle. We give an example of this stable cycle with its basin in Figure 3f. Regarding cycles of period 5, the behavior of the map (5) changes. Figure 3g shows the region in the 2D bifurcation diagram where we can get this cycle. Numerical simulation shows that not all those points give a stable period 5-cycle from the beginning. Some of these points give a stable period 5-cycle, and the others give chaotic behavior at the beginning, which then turns to a stable period 5-cycle. To clarify, let us start with the following parameter values: m=0.7372 and b=0.4312. Figure 3h presents the basin of attraction of the period 5-cycle. This cycles is not stable from the beginning. The map starts chaotically and then turns to a stable period of cycle 5, as shown in the time series in Figure 3i. On the other hand, if we take m=0.9503 and b=0.2485, we get a stable period 5-cycle from the beginning, as shown in Figure 3j. Figure 3k shows the period 6-cycle in the 2D bifurcation diagram. Interestingly, this region includes points for the two parameters, *m* and *b*, where period 3-cycle also exists with the period 6-cycle. In addition, it contains some points for those parameters where chaotic behavior can be obtained. For example, we take m=0.7133 and b=0.4528 from this region, defined in Figure 3k. We get a stable cycle of period 3. It is given in Figure 3l with its basin of attraction. But if we assume that m=0.7779 and b=0.3598, we get a chaotic situation in the map (5) that turns to a stable period of cycle 6 when time increases. Interested readers are advised to investigate the other regions in the 2D bifurcation diagram to get more interesting results about that map. Therefore, we end this section with this analysis in order to reduce the number of figures in this paper; however, for example, the region for period 7-cycles includes points that give other types of cycles, such as period 14-cycles, 24-cycles, and chaotic behavior.

#### 3.2.2. Chaotic Attractor

**Figure 4:**
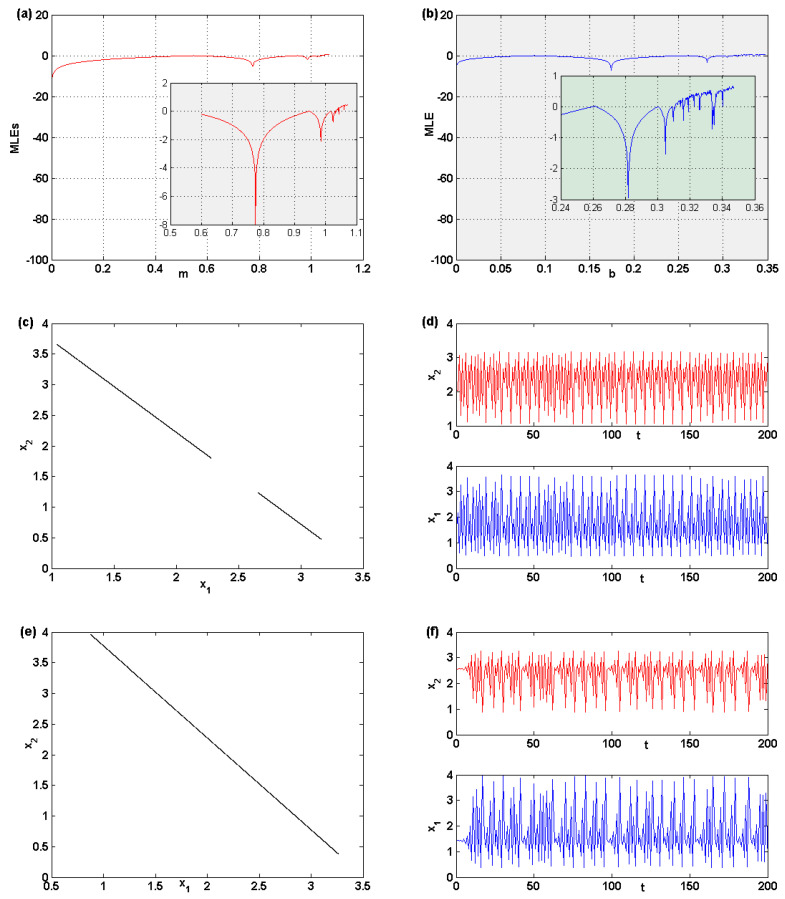
(**a**) Maximum Lyapunov exponent when varying the parameter *m*. (**b**) Maximum Lyapunov exponent when varying the parameter *b*. (**c**) A two-piece chaotic attractor. (**d**) Time series for the two-piece chaotic attractor. (**e**) One piece chaotic attractor. (**f**) Time series for the one piece chaotic attractor.

The numerical simulation shows that both parameters, *m* and *b*, are responsible for the chaotic behavior of the map (5). More studies are carried out here in this part to investigate the characteristics of this map more. Maximum Lyapunov exponents (MLEs) are important in discovering such characteristics. They provides evidence that the behavior of the map enters the chaotic region. Assuming that b=0.2,p=0.3, and q=0.2, the MLE for the parameter *m* is given in Figure 4a. It shows that the map enters a chaotic region when varying that parameter. The figure shows that, when increasing *m*, the distribution of MLE changes from negative to positive values, which is evidence of chaotic behavior in the map. The same observation is given in Figure 4b for the parameter *b* when fixing the parameter values to m=0.83,p=0.3, and q=0.2. This urges us to detect the chaotic attractor of the behavior of the map. Let us assume that m=1.0445,b=0.2,p=0.3, and q=0.2; besides that, we assume some initials, $x_{1,0}=2.04$ and $x_{2,0}=1.434$, that lie close to the fixed point $e_{2}$. This assumption gives birth to a chaotic attractor in two pieces. It is plotted in Figure 4c, and the time series for both variables are given in Figure 4d. Increasing *m* to the value 1.055, the evolution of the map gives birth to a one-piece chaotic behavior, as depicted in Figure 4e with the time series given in Figure 4f. Several numerical experiments were carried out and showed that any other increase in the parameter *m*, with keeping the other parameter values fixed, gives only one piece of chaotic attractor. The same observations can be obtained from the other parameter *b*.

#### 3.2.3. Entropy Analysis

In statistics, there are different measures that can be used to investigate time series data. Of these measures, there is the approximated entropy (ApEn) technique. This technique is used to measure regularity and predictability over time series data. It is important to mention here that exact entropy cannot be experimentally calculated. For this reason, we instead calculate the approximated entropy. It is a way of measuring or quantifying level of chaos in chaotic systems. In literature [21,22], there are many algorithms that have been proposed to investigate the level of chaos in discrete dynamic systems. In this paper, we recall the algorithm of approximated entropy in Reference [21] to do our experiments. The following table Table 1 gives the ApEn for different values of the parameters *m* and *b*. It is observed that, when the ApEn is very small (last row in the following table), both of the time series are regular and predictable (the case of 5 cycle). The other ApEn calculated values in the table represent a chaotic behavior of the map.

## 4. Discussion

Considering a kind of preference that depends on past choice and not constant, as studied in literature, gives rise to interesting outcomes using numerical experiments. The obtained results show that, whether or not the fixed points of the economic models stable, some interesting complex dynamic characteristics exist. There may be dangerous situations, such as zero or negative consumption of goods. This leads to assuming positive values for the consumption of goods in the proposed economic models in order to enlarge the region in the feasible space which contains high periodic points. We have shown that those periodic cycles with positive values attract all the points in the phase space. Furthermore, the assumption of non-constant preferences in the utility function gives some restrictions on the experience-dependent parameter *b*. The numerical experiments shows that any increase in this parameter gives more swing in favor of one good against the other. In addition, assuming negative values for this parameter should not be assumed; otherwise, dangerous situations, such as negative consumption of goods, can be obtained.

## 5. Conclusions

This paper studied the dynamics of a game with players using quantities as their decision variables, and their preferences depended on past choices. By assuming such preferences, a discrete dynamic map was obtained. The conditions of stability of fixed points of this map was analyzed. The results showed that those points lose their stability due to fold and flip bifurcation. We also discussed the existence of different types of periodic cycles and chaotic behaviors that arise due to those types of bifurcations, as well as gave some entropy analysis for certain behaviors of the game’s map.

## Figures and Tables

**Table 1:** ApEn of the map (5) for different values of the parameters *m* and *b*.

$x_{0,1}$	$x_{0,2}$	*m*	*b*	ApEn (Series) $x_{1}$	ApEn (Series) $x_{2}$	Figure
2.04	1.434	1.044	0.2	0.0741	0.0657	Figure 4d
2.56	1.42	1.055	0.2	0.1575	0.1315	Figure 4f
2.57	1.41	1.057	0.2	0.1794	0.1286	chaotic attractor
2.58	1.41	1.059	0.2	0.00022572	0.00022572	cycle 5
